# Self-management of patients with tracheostomy in the home setting: a scoping review

**DOI:** 10.1186/s41687-023-00643-2

**Published:** 2023-10-12

**Authors:** Sandra Weidlich, Jens Pfeiffer, Christiane Kugler

**Affiliations:** 1 Faculty of Medicine, Department of Oto-Rhino-Laryngology, Medical Center - University of Freiburg, Freiburg, Germany; 2 Center for Oto-Rhino-Laryngology (HNO Center am Theater), Freiburg, Germany; 3 Faculty of Medicine, Institute of Nursing Science, University of Freiburg, Breisacher Str. 153, Freiburg, 79110 Germany

**Keywords:** Self-management, Tracheostomy, Laryngectomy, Tasks, Skills

## Abstract

**Purpose:**

The aim of this study was to create a model of patient-centered outcomes with respect to self-management tasks and skills of patients with a tracheostomy in their home setting.

**Methods:**

A scoping review using four search engines was undertaken (Medline, CINAHL, PsycINFO, Cochrane Library) to identify studies relevant to this issue and published since 2000. The Preferred Reporting Items for Systematic Reviews and Meta-Analyses Statements for Scoping Reviews (PRISMA-ScR), the Joanna Briggs Institute (JBI) approach of conducting and reporting a scoping review, and the Participants, Concept, Context (PCC) scheme were employed. The following elements of the framework synthesis study data were screened, and presented based on the self-management model of Lorig and Holman.

**Results:**

34 publications from 17 countries met the criteria for study inclusion: 24 quantitative, 8 qualitative and 2 mixed methods designs. Regarding the dimensions of self-management, 28 articles reported on “managing the therapeutic regimen”, 27 articles discussed “managing role and behavior changes”, and 16 articles explored “managing emotions”. A model of self-management of patients with tracheostomy was developed, which placed the patient in the center, since it is this individual who is completing the tasks and carrying out his or her skill sets.

**Conclusion:**

This scoping review represents the first comprehensive overview and modeling of the complex self-management tasks and skills required of patients with tracheostomy in their home setting. The theoretical model can serve as a cornerstone for empirical intervention studies to better support this patient-centered outcome for this population in the future.

**Supplementary Information:**

The online version contains supplementary material available at 10.1186/s41687-023-00643-2.

## Background

Although about 250,000 tracheostomies are performed annually worldwide in resource rich countries [1], living with a tracheostomy is far from normal. Those affected have to adjust to a wide range of changes to manage their day-to-day life [2–4]. The term tracheotomy refers to an incision in the trachea; tracheostomy represents a temporary or permanent opening in the neck [5, 6]. However, the terms for the procedure are often used interchangeably [5]. A tracheostoma describes an opening into the trachea. Through the stoma a tracheostomy tube is usually inserted [5]. Nowadays, a tracheotomy is performed as an elective as well as an emergency surgical procedure. Indications have expanded over time [7], the most prominent being to provide mechanical ventilation, to protect the airway, and to bypass an upper airway obstruction [5, 6]. In particular, the total laryngectomy should be mentioned in this context, the major treatment modality for stage III-IV laryngeal cancer [8], which requires the surgical removal of the larynx and leads to a permanent artificial airway which is separate from mouth, nose, and esophagus.

Because of the varying indications the impacted patient population ranges from the critically ill patient with intensive care requirements to the independent patient who has received a tracheostomy as part of the medical therapy [9]. Consequently, care of patients with tracheostomy takes place in different healthcare settings, both in the hospital and in outpatient settings [5]. Due to a decline in hospital length of stay, there is an increasing percentage of patients with tracheostomy living in the community [10]. In Germany it is assumed that around 15,000–30,000 patients are affected [11]. In the hospital as well as in the outpatient environment consistent care of patients with tracheostomy is emphasized [5]. However, patients report many negative experiences [7], which may impact their quality of life [7, 12–16].

A publication by Richard and Shea (2011) delineated self-care and associated concepts, according to them self-care, self-management, self-monitoring, and symptom management represent overlapping concepts that build on each other [17]. However, a consensus on the definition of the concept does not exist [18]. Self-management is an overarching concept that includes all “[…] activities necessary to achieve, maintain, or promote optimal health […]” [17, p 261]. It represents the ability of the individual to manage the consequences of health conditions [17]. Following Richard and Shea [17], and Matarese and associates [19], self-management of patients with tracheostomy is conceptualized as the individual’s ability to perform activities related to the care of a tracheostomy and to cope with the life impact of the presence of a tracheostomy. According to Lorig and Holman [20], self-management comprises three dimensions: managing the therapeutic regimen; managing role and behavior changes; and managing emotions. This encompasses self-management tasks and the development of core self-management skills including problem-solving, decision-making, resource utilization, forming a partnership with the healthcare provider, and taking action [20]. Self-management provides opportunities for patients to positively impact their health and health-related habits [21]. Patients are supported in gaining skills and applying them to their routines on a daily basis [22]. Self-management programs have been developed for specific patient populations and have been evaluated as successful in terms of health outcomes and costs [21, 23].

Patients with a tracheostomy are required to manage a comprehensive therapeutic regimen including cannula cleaning, stoma care and dressing changes [24] in order to avoid complications, which can be life-threatening [9]. Daily tracheostomy care, which includes the use of a variety of assistive devices, is very time-consuming [12, 25] and associated with fears, uncertainties [26], and restrictions in numerous activities of daily living [27]. Furthermore, several studies report reduced general health in these patients [28–30]. The presence of a tracheostomy leads to physical changes, especially with regard to communication, breathing and nutrition [3, 7, 31]. Moreover, those individuals have to adapt to a changed body image [3, 31]. In a study with patients after laryngectomy, half of the respondents felt embarrassed [32]. They feel stigmatized and isolated [7]. They withdraw from social interactions [32, 33] which influences their social relationships [33]. Psychological effects [34] and a decline in mental health [30] also are described. Patients reported higher levels of depression and anxiety compared to the normative population [28]. Considering the complex impact the tracheostomy has on a patients’ life, those affected have to learn tasks [34] and skills [35] to care for and live with their tracheostomy. A focus on patient-centered outcomes becomes increasingly important, and successful self-management of the tracheostomy in their home setting is needed.

To the best of our knowledge, there is no review of patient-centered outcome research with respect to self-management for patients with tracheostomy. An overview of self-management tasks and skills for patients with tracheostomy could help to guide the development of population specific interventions, their implementation and evaluation, with the aim of improving patient quality of life. Thus, the aim of this paper was to review the literature in order to gain an understanding of patient self-management when living with a tracheostomy at home.

## Methods

For this purpose, a scoping review was conducted [36–38]. Evidence gaps were identified. This review was conducted and reported in concordance with the Preferred Reporting Items for Systematic Reviews and Meta-Analyses Statements for Scoping Reviews (PRISMA-ScR) [39] and the Joanna Briggs Institute (JBI) approach to conducting and reporting scoping reviews [40].

### Literature search

After a limited search in Medline and CINAHL to identify keywords and index terms the final search in the databases Medline, CINAHL, PsycINFO, and Cochrane Library was conducted with support by a librarian. Due to a paradigm shift from paternalistic towards patient-centered care the concept of self-mnagement emerged and has been accompanied by an increasing number of publications in this field [41]. Consequently, articles with a publication year of 2000 or later were included in the search for this review. A free text and keyword search was performed using the PCC scheme with search terms related to the participants (P) “patients with tracheostomy”, the concept (C) “self-management” (including related concepts due to their inconsistent use), and context (C) “home setting”. Available Medical Subject Headings (MeSH) terms were added. Search terms were logically combined. Please see supplementary file 1 as an online resource. The process followed the flowchart for study selection adapted from the PRISMA statement [42].

### Inclusion and exclusion criteria

Inclusion and exclusion criteria were defined using the PCC scheme (Participants, Concept, Context) [36]. All records that fulfilled the following criteria and were available in English or German language were included:

#### Participants (P)

Studies of adults (> 18 years of age) either with a temporary or permanent tracheostomy who were fully or partially self-sufficient in tracheostomy care were included. Research on ventilated patients was excluded due to their additional and possibly other needs in managing their self-management.

#### Concept (C)

This review considered studies that described aspects of self-management in the context of tracheostomy care from the point of view of those affected. Following Richard and Shea [17], and Matarese et al. [19] self-management involved the ability of an individual to perform actions related to the care of a tracheostomy as needed and to deal with the changes in lifestyle and the effects associated with the tracheostomy. For further operationalization self-management tasks and skills included in self-management processes [43] were considered following the model of Lorig and Holman [20].

#### Context (C)

Studies taking place in the home setting were considered. Research studies that examined the acute inpatient, rehabilitative or nursing home setting as well as publications that focused on tracheostomy care in the context of end-of-life (understood as expected life expectancy of a few months [44]) were excluded. Studies that took place in less developed countries, as well as low and middle income countries (DAC list) [45], were also purposefully excluded because external factors such as available resources, environmental factors, and the health care system may have influenced self-management [21, 46].

This scoping review included qualitative, quantitative, mixed methods studies and systematic reviews. Case studies, text and opinion papers, and letters (editorials, commentaries) were not considered.

### Data abstraction and analysis

Data from the included articles were abstracted applying elements of framework synthesis, a strategy for collecting data in scoping reviews [40]. It aims to reduce complexity [47] by searching, screening, and presenting study data using a pre-identified concept [40, 47]. Coding categories were established based on pre-identified themes and expanded with themes that emerged from the data [48]. Based on the self-management model by Lorig and Holman [20] the following dimensions were selected to code data in this review: managing the therapeutic regimen; managing role and behavior changes; and managing emotions [20]. After assigning content to the appropriate dimension, sub-dimensions were created if useful. Each process step was performed by one person, after each separate step the work group met to discuss, critically appraise and reach consensus with regards to findings from this step before the person performing this study moved forward to the next step within this process. Study characteristics were displayed in tabular format.

## Results

**Fig. 1:**
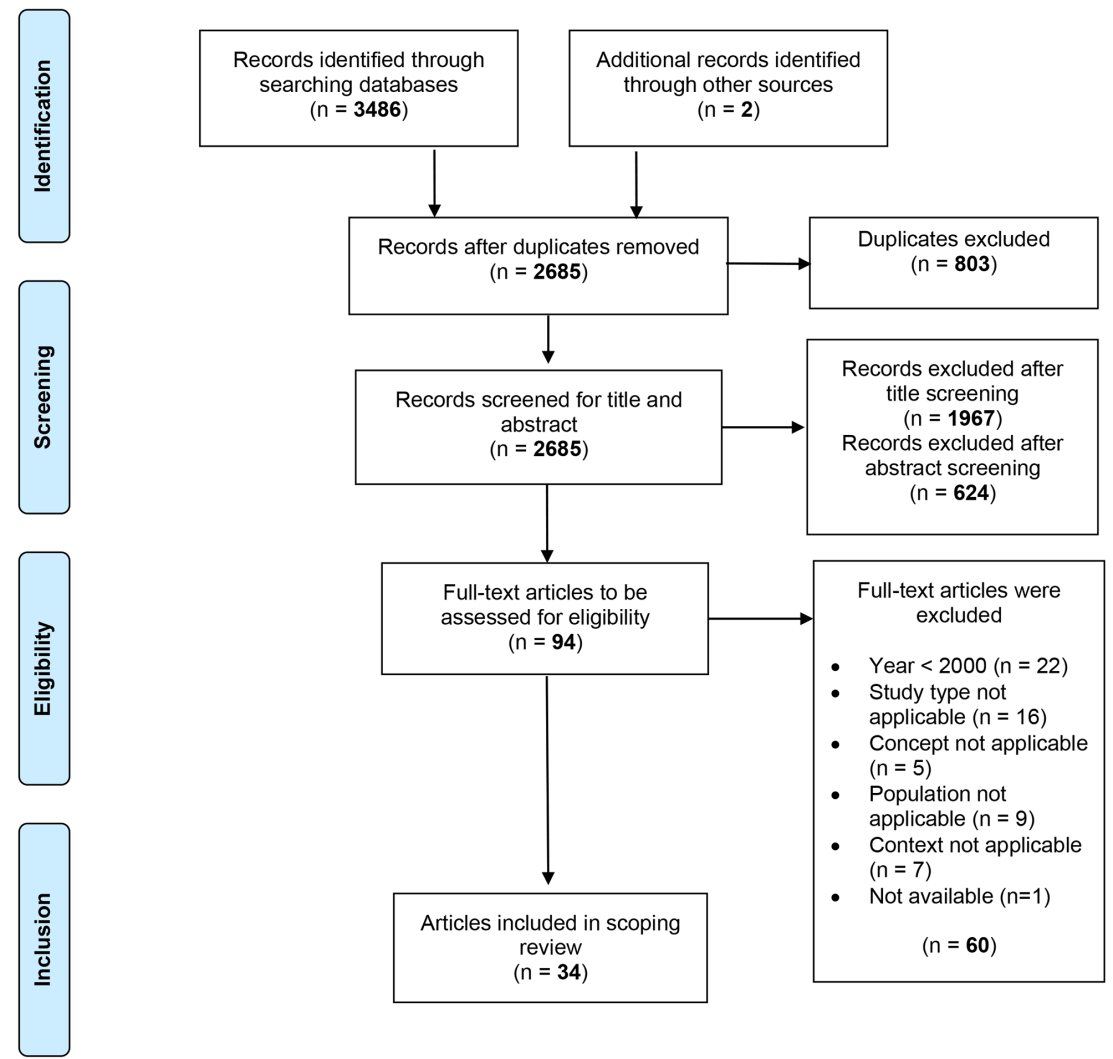
Flow chart of the search process according to PRISMA 2009 [41]

The search in Medline, CINAHL, PsycINFO, Cochrane Library resulted in 3,488 records. After removing duplicates, 2,685 articles remained. After title and abstract screening, 94 full texts were assessed for eligibility. Finally, 34 studies met the criteria for inclusion. The selection process is illustrated in Fig. 1 based on the PRISMA statement as recommended by Moher et al. [42].

### Study characteristics

Overall, 34 publications with a focus on patient-centered outcomes from 17 countries, published in the period of the literature search, were included. Study designs captured were quantitative (n = 24; 71%), qualitative (n = 8; 23%), and mixed methods (n = 2, 6%). The objectives of the studies varied. In 47% of quantitative studies (n = 16; 47%) quality of life was the major focus, whereas in 18% of qualitative studies experiences (n = 6; 18%), in one qualitative study adjustment (n = 1; 3%), in one qualitative study needs (n = 1; 3%), and in one qualitative study body image (n = 1; 3%) were the focus of the research. Time from tracheostomy surgery to data collection varied considerably. In 71% of all included studies tracheostomy surgery was on average more than six months prior to data collection (n = 24; 71%). Three studies (9%) utilized longitudinal designs with data collection at six months [49], one year [29], and three years [50] after tracheostomy surgery. 91% of all included publications studied patients after laryngectomy (n = 31; 91%), patients with a permanent artificial airway created after surgical removal of the larynx and which is separate from mouth, nose and esophagus. For more details on the study characteristics see Table 1.

**Table 1:** Descriptive overview of study characteristics

Study	Study Design	Study Objective	Study Population		
			Time since tracheostomy	Sample size (n)^a^	Type of tracheostomy / Indication of tracheostomy
Wulff et al. [16]; 2021; Denmark, Sweden	Quantitative, Cross-sectional study	HRQoL, voice problems, dysphagia, depression, anxiety	1.6–18.1 years (median 6,3 years)	172	LE
Sluis et al. [51]; 2020; Netherlands	Qualitative, interpretative phenomenological	Experiences of women	Range 1–31 years	8	LE
Bickford et al. [35]; 2019; Australia	Qualitative, Grounded Theory	Experiences	2–11 years	12 (+ 9 primary supporters, 7 health professionals)*	LE
Teruya et al. [52]; 2019; Japan	Quantitative, Cross-sectional study	Acceptance, Daily life difficulties	Ø 6.3 ± 6.5 years	43	LE
Bickford et al. [53]; 2018; Australia	Qualitative, Grounded Theory	Adjustment to physical and functional changes	2–11 years	12 (+ 9 primary supporters, 7 health professionals)^b^	LE
Jansen et al. [54]; 2018; Netherlands	Quantitative, Cross-sectional study	Unmet supported care needs	Median 7 years (range 0–37 years)	283	LE
Mertl et al. [25]; 2018; Czech republic	Mixed methods study	QoL, stigmatization, social exclusion	(preop), 4–6 months	22 (quantitative), 6 (qualitative)	LE
Morris et al. [55]; 2017; USA	Qualitative, approach of Naturalistic inquiry	Body image perception	≥ 1–2 months, not specified	36	Not specified, even LE
Cnossen et al. [56]; 2016; Netherlands	Qualitative, Focus group interviews	Needs assessment	Ø 11,6 years (range 2–22)	9 (+ 3 partners)	LE
Offerman et al. [57]; 2015; Netherlands	Quantitative, Cross-sectional study	Spousal relationship	< 1 year (10%), 1–10 years (52%), > 10 years (38%)	151 (+ 144 partners)	LE
Pereira et al. [12]; 2015; Portugal	Quantitative, Cross-sectional study	QoL	Ø 37,18 months	34	LE
Perry et al. [28]; 2015; Australia	Quantitative, Cross-sectional study	QoL	< 1 year (11%), > 1 year (89%)	86	LE
Roick et al. [58]; 2014; Germany	Quantitative, Cross-sectional study	QoL, Social Integration	1 year	161	LE
Singer et al. [29]; 2014; Germany	Quantitative, Cohort study, Longitudinal study	QoL	Period: preop – 1 year	174	LE
Bickford et al. [2]; 2013; Australia	Qualitative, Grounded Theory	Experiences	2–11 years	12	LE
Singer et al. [50]; 2013; Germany	Quantitative, Cohort study, Longitudinal study	Vocational Rehabilitation	Period: preop – 3 years	231	LE
Dooks et al. [3]; 2012; Canada	Qualitative, Interpretative	Experiences of reintegration into the community	6–12 month	9	LE
Tsikoudas et al. [59]; 2011; Great Britain	Quantitative Case-control study	QoL, nasal function	Range: 7 days – 11 years	IGr = 10, CGr = 10	Head and neck tumor (not specified)
Danker et al. [32]; 2010; Germany	Quantitative, Cross-sectional study	Social withdrawal	Ø 6 years (range 0–18 years)	219	LE
Hashmi et al. [30] 2010; USA	Quantitative, Cohort study	QoL, self-image	prospective: (preop), 1–3 weeks; retrospective: > 6 month	7 (prospective), 6 (retrospective)	Bilateral vocal fold / cord paralysis, benign laryngeal tumor, laryngeal / laryngeotracheal stenosis, sarcoidosis
Noonan et al. [60]; 2010; Ireland	Qualitative, semi-structured interviews	Experiences	Range 1,5–7 years	10	LE
Babin et al. [33]; 2009; France	Quantitative, Cross-sectional study	Psychosocial QoL changes	Ø 6 month (median 4 years)	150	LE
Minovi et al. [61]; 2009; Germany	Quantitative, Cross-sectional study	QoL	56,3 ± 28,1 months	30	LE
Singer et al. [62]; 2007; Germany	Quantitative, Cross-sectional study	Use of adaptive devices	Ø 6 years (range 1–26 years)	218	LE
Singer et al. [63]; 2007; Germany	Quantitative, Cross-sectional study	Stigmatization	Ø 6,4 years (1–20 years)	217	LE
Woodard et al. [64]; 2007; USA	Quantitative, Cohort Study	QoL	Not specified	33	LE
Vilaseca et al. [65]; 2006; Spain	Quantitative, Cross-sectional study	QoL	Ø 9.09 years (range 2–29 years)	49	LE
Gilony et al. [13]; 2005; Israel	Quantitative, Cross-sectional study	QoL, well-being, body-image	≥ 3 month, not specified	24	Nonmalignant condition
Schuster et al. [66]; 2003; Germany	Quantitative, Cross-sectional study	Coping strategies	Ø 4 ± 3,6 years (range 9 months – 17 years)	25	LE
Birkhaug et al. [67]; 2002; Norway	Quantitative, Cross-sectional study	QoL, mood level	10 ± 7 years	104	LE
Armstrong et al. [49]; 2001; Australia	Quantitative, Longitudinal study	physical, psychological, and social problems	Period: preop − 6 month (assessments pre-op, and at 1, 3, 6 mts post-op)	34	LE
Lennie et al. [68]; 2001; USA	Mixed methods study	Eating related experiences	Ø 5 years (range 0,25–16 years)	34	LE
Nalbadian et al. [4]; 2001; Greece	Quantitative, Cross-sectional study	QoL	7–120 months (median 43 months)	56	LE
Relic et al. [69]; 2001; Germany	Quantitative, Cross-sectional study	QoL	Ø 6 years (range 1–11 years)	29	LE

Abbreviations: Ref. = Reference number; QoL = quality of life; HRQoL = Health related quality of life; Ø = on average; preop = preoperative; IGr = intervention group; CGr = control group; LE = laryngectomy

^a^ Baseline data are presented.

^b^ For the purpose of this study, data from the patient sub-sample have been used.

### Self-management of patients with tracheostomy

The studies described multiple changes in lifestyle when living with a tracheostomy in the home setting. Regarding the dimensions of self-management, 28 articles (82%) discussed management of the therapeutic regimen, 27 articles (79%) examined managing role and behavior changes, while 16 articles (47%) described managing emotions. An overview of the captured self-management dimensions in the included studies are given in Table 2. Table 3 displays the dimensions and sub-dimensions of self-management of patients with tracheostomy.

**Table 2:** Overview of captured self-management dimensions and sub-dimensions

Study	Managing the therapeutic regimen		Managing role & behavior changes		Managing emotions	
	Tracheostomy care	Tracheostomy-related physical/functional changes	Changed roles	Changed everyday behavior	Negative emotions	Positive emotions
Wulff et al. [16]; 2021; Denmark, Sweden		√	√	√	√	
Sluis et al. [51]; 2020; Netherlands	√	√	√	√	√	
Bickford et al. [35]; 2019; Australia		√	√	√		
Teruya et al. [52]; 2019; Japan		√		√		
Bickford et al. [53]; 2018; Australia		√	√	√	√	
Jansen et al. [54]; 2018; Netherlands		√		√		
Mertl et al. [25]; 2018; Czech republic	√	√	√	√	√	
Morris et al. [55]; 2017; USA		√		√	√	√
Cnossen et al. [56]; 2016; Netherlands	√	√		√		
Offerman et al. [57]; 2015; Netherlands		√	√	√	√	
Pereira et al. [12]; 2015; Portugal		√			√	
Perry et al. [28]; 2015; Australia			√		√	
Roick et al. [58]; 2014; Germany				√		
Singer et al. [29]; 2014; Germany		√		√		
Bickford et al. [2]; 2013; Australia		√	√	√	√	√
Singer et al. [50]; 2013; Germany		√	√			
Dooks et al. [3]; 2012; Canada	√	√	√	√	√	
Tsikoudas et al. [59]; 2011; Great Britain		√			√	
Danker et al. [32]; 2010; Germany		√	√	√	√	
Hashmi et al. [30]; 2010; USA					√	
Noonan et al. [60]; 2010; Ireland		√		√	√	√
Babin et al. [33]; 2009; France				√		
Minovi et al. [61]; 2009; Germany		√				
Singer et al. [62]; 2007; Germany	√					
Singer et al. [63]; 2007; Germany				√	√	
Woodard et al. [64]; 2007; USA		√	√	√		
Vilaseca et al. [65]; 2006; Spain		√				
Gilony et al. [13]; 2005; Israel		√				
Schuster et al. [66]; 2003; Germany		√	√	√		
Birkhaug et al. [67]; 2002; Norway			√			
Armstrong et al. [49]; 2001; Australia	√	√	√	√		
Lennie et al. [68]; 2001; USA		√		√		
Nalbadian et al. [4]; 2001; Greece		√	√	√	√	
Relic et al. [69]; 2001; Germany		√	√	√		
Total	6	27	17	24	16	3
	28 (82%)		27 (79%)		16 (47%)	

**Fig. 2:**
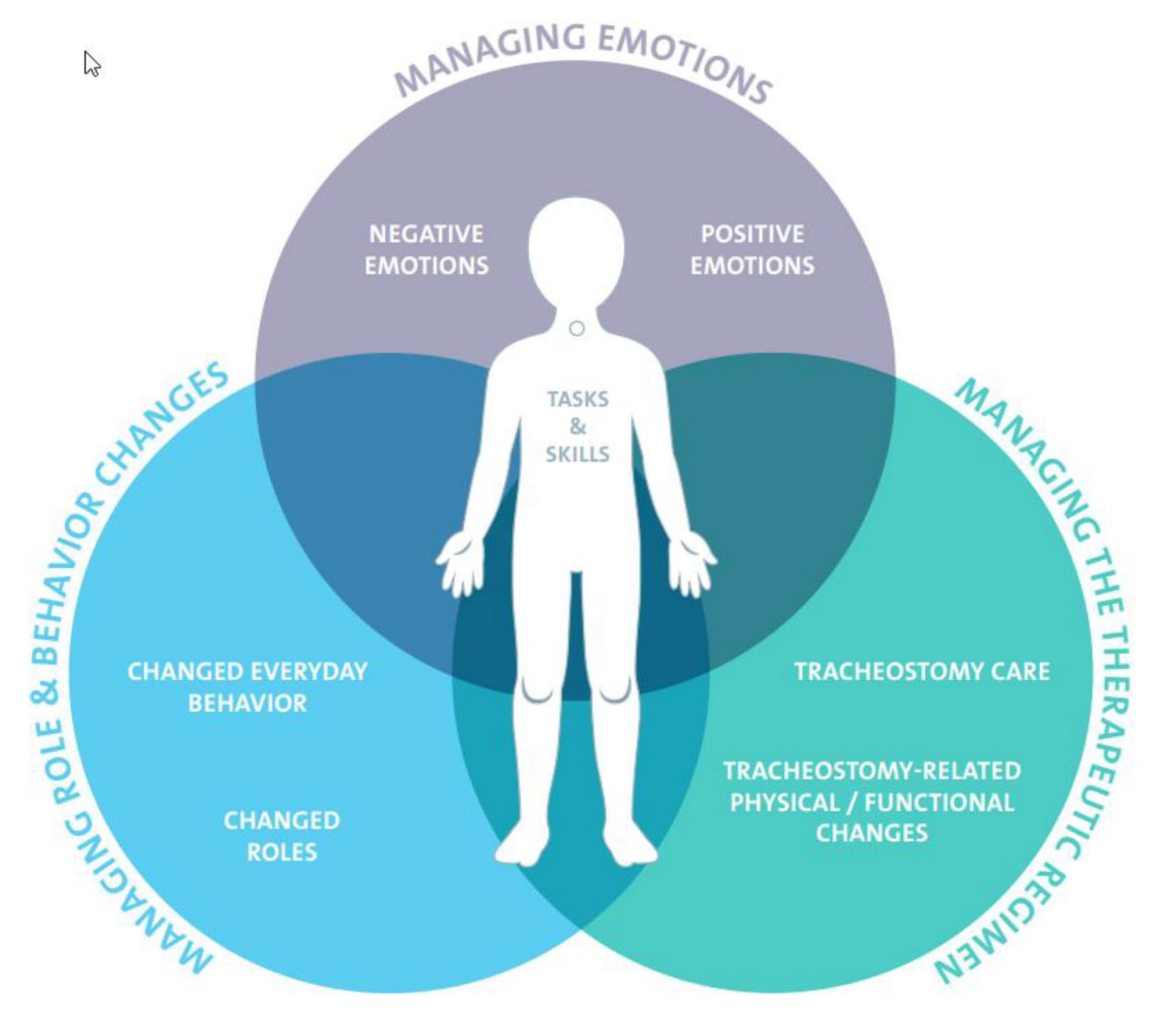
Model of self-management of patients with tracheostomy

Patients with tracheostomy are challenged by a complex therapeutic regimen. In concordance with the generic self-management model provided by Lorig and Holman [20], three self-management dimensions were identified for patients with tracheostomy by this review. Based on these findings, a model of self-management of patients with tracheostomy was developed (Fig. 2).

The patient with a tracheostomy was placed in the center of this pictogram because it is this individual who must perform the tasks and apply his or her skill sets needed for self-management. The circles surrounding the individual represent the self-management dimensions of patients with tracheostomy following Lorig and Holman [20]. Each dimension can be divided into sub-dimensions based on the results of this scoping review. The dimensions overlap based on to the studies included. Detailed information can be seen in Table 4.

**Table 3:** Self-management of patients with tracheostomy

Self-management		
Dimension	Sub-dimension^a^	Managing the impact of
**Managing the therapeutic regimen**	**Tracheostomy care** (n = 6)	• **Altered airway** [3] leads to *protecting the stoma in cold or dusty environment* [3] • **Stoma care** [56] • **Complications** [49] • **Medical aids** [51] such as tracheostomy tube [62], communication aids [62], voice prothesis [56], humidification aids [62], suction devices [62], shower / swimming [62], others (e.g. jewelry) [62] • **Altered body care** regarding dealing with water [25], showering [3, 51] leads to the need to *protecting the stoma* [3]
	**Tracheostomy-related physical/** **functional changes** (n = 27)	• **Altered speech** [2, 3, 16, 29, 35, 49, 56, 51, 54, 64, 65, 69] and **altered voice** [4, 16, 25, 32, 51, 53] lead to the need to *learn to speak* [50], *to adopt to new speaking methods* [3] by *using communication aids* [49] / *technology* [3] • **Altered respiratory function** [2, 52] / respiratory problems [25] regarding altered secretion / bronchial discharge [3, 4, 60], cough [3, 16, 69], dyspnea [16, 29], noisy breathiness [13], shortness of breath [54] lead to the need to *managing mucus discharge* [3, 51] • **Altered swallowing** [2, 16, 49, 56, 51, 54, 60] leads to the need to *careful eating* [60] • **Altered senses** [16, 29], altered smell [3, 4, 25, 49, 54, 59, 61, 68], altered taste [3, 25, 49, 54, 59, 68] • **Altered oral health** regarding dry mouth [54, 61, 66], sticky saliva [16, 54, 61] • **Pain** in the shoulder, neck [49, 56, 66], when swallowing [66] • **Altered defecation** [52] • **Altered appearance** [2, 4, 25, 35, 51, 65] associated with (dis)figurement [57], aesthetics [64] and altered body image perception [3, 13, 55] • **Power(lessness)** [25, 54, 60], altered capacity [12, 49], physical fitness [51] and activity [65] leads to the need to *improving physical performance* [50] and *recovery* [50] • **Fatigue** [29, 69], tiredness [54]
**Managing** **role & behavior changes**	**Changed roles** (n = 17)	• **Altered self-identity** [25, 35, 53]: loss of femininity [51], being a man / woman [57], gender confusion [2] • **Altered social life** [49] regarding social roles [28, 35, 51, 53], family [2–4, 51, 69], (spousal) relationship [49, 51, 57], friendships [35], sexual life [4, 16, 51, 57] leads to social disruption [64], *searching for social integration, information and exchange of experience* [66] by *developing new roles* [2] and *getting to know other patients* [50], for example *active membership* [32, 67] in *peer groups* [3] • **Altered professional life** [2, 3, 50, 51, 53] leads to the need to *occupational rehabilitation* [50] and/or *starting work-related activities* [51]
	**Changed everyday behavior** (n = 24)	• **Altered social participation** [2, 53, 58] in social activities [3, 4, 29, 32, 33] regarding social eating / eating out in public [3, 16, 32, 33, 49, 54, 53, 68], events [32, 33] lead to the need to *adjusting to social settings* [55] / *daily situations* [2], *social withdrawal* [16, 51, 63] • **Altered sports activities** [25, 33] **/ leisure activities** [33, 51] lead to the need to *adjustments to perform hobbies* [51], *take new activities* [33, 51], *resume activities* [35, 51] • **Altered communication** [2, 32] with partners [57] and others [4, 52], also phone calls [3, 4, 69] lead to *communication in different ways* [32] • **Altered eating** [35, 56, 54, 52, 60, 64] regarding altered enjoyment [68, 69], appetite [29, 66], time required [68]
**Managing emotions**	**Negative emotions** (n = 16)	• **Distress** [3, 12, 25, 32], depression [2, 4, 16, 25, 28, 32, 55, 60, 63], feeling of uselessness [25], feeling of loneliness [4], feeling of vulnerability [3], concern [55] • **Fear /** anxiety [16, 32] for breathing problems [28, 51, 57, 63], restlessness [59] • **Anger** (frustration [3, 59], irritability [59]) • **Shame** [3, 4, 32, 57], worsened self-esteem [30] leads to *cover tracheostomy* [2] *by clothing selection* [53] • **Guilt** (sense of regret [3, 60]) • **Stigma** [3, 25, 32, 51, 55, 63] leads to the need to *deal with negative reactions of others* [55]
	**Positive emotions** (n = 3)	• **Life saving** feeling [2, 55] • **Optimism** [60]

Note: The dimensions based on Lorig and Holman [20]. The sub-dimensions are based on the results of this scoping review. For better readability generic terms have been highlighted. The order is valueless.

^a^ Studies included have mostly taken more than one self-management aspect into account.

**Table 4:** Overlapping of dimensions / sub-dimensions

Study	Managing the therapeutic regimen		Managing role & behavior changes		Managing emotions
	Tracheostomy care	Tracheostomy- related physical/ functional changes	Changed roles	Changed everyday behavior	Negative emotions
Sluis et al. [51]; 2020; Netherlands		Altered voice	Altered self-identity: loss of femininity Altered social life: family		
		Altered appearance			Stigma
Bickford et al. [35]; 2019; Australia		Altered speech Altered appearance	Altered self-identity Altered social life: social roles	Altered eating	
Bickford et al. [53]; 2018; Australia		Altered voice	Altered self-identity Altered social life: social roles Altered professional life	Altered social participation: social eating/ eating out in public	Shame: clothing selection
Mertl et al. [25]; 2018; Czech republic		Altered appearance			Stigma
		Altered voice	Altered self-identity		
Offerman et al. [57]; 2015; Netherlands		Altered appearance: disfigurement	Altered social life: sexual life		Shame Fear / anxiety for breathing problems
Dooks et al. [3]; 2012; Canada	Altered airway: protecting the stoma in cold or dusty environment	Altered respiratory function: secretion / bronchial discharge, cough		Altered social participation: social eating / eating out in public	Shame
		Altered speech			Anger: frustration
		Altered appearance: body image		Altered social participation in social activities: social eating / eating out in public	Stigma
Danker et al. [32]; 2010; Germany		Altered voice		Altered social participation in social activities	Distress, depression Stigma Fear / anxiety
Singer et al. [63]; 2007; Germany				Altered social participation: social withdrawal	Stigma
Armstrong et al. [49]; 2001; Australia		Altered speech Altered swallowing		Altered social participation: social eating /eating out in public	
Lennie et al. [68]; 2001; USA		Altered senses: smell, taste		Altered social participation: social eating / eating out in public	

#### Dimension 1: managing the therapeutic regimen

Six publications (18%) reported on self-management in the context of ***tracheostomy care***, which means managing an altered airway [3] and includes stoma care [56], dealing with complications [49] and using medical aids [51]. The latter includes products that are necessary for living with a tracheostomy: tracheostomy tube [62], humidification aids [62], suction devices [62], communication aids [62], and voice prostheses [56] for laryngectomy patients as well as aids that (should) make life easier (such as aids to shower and swim, jewelry to cover the tracheostomy [62]). Moreover, patients have to manage an altered body care concerning dealing with water [25] and showering [3, 51]. Twenty- seven studies (79%) described aspects of managing the ***tracheostomy- related physical/ functional changes***. In this context, alterations in speech / voice, respiratory function, swallowing, senses, oral health, pain, defecation, appearance, power(lessness) and fatigue, including associated tasks, were identified.

#### Dimension 2: managing role and behavior changes

This portion of the model included studies that described changed roles and everyday behavior which have to be managed by patients. Seventeen studies (50%) reported ***changed roles***. In this context changes in self-identity, social and professional life were reported. Twenty- four studies (71%) described ***changed everyday behavior***. These changes related to social participation, sports activities / leisure activities, communication and eating.

#### Dimension 3: managing emotions

Sixteen studies (47%) reported on emotions after tracheostomy, patients had to manage in their home setting. The management of ***negative emotions*** were described in sixteen studies (47%). According to Izard [70, 71] changes in emotions were related to the concepts of distress, fear, anger, shame, and guilt, stigma was related to these concepts. Three studies reported on ***positive emotions*** and included life-saving feeling and optimism.

## Discussion

In this comprehensive scoping review, thirty-four articles on patient-centered outcomes with respect to self-management in patients with a tracheostomy in their home setting were synthesized. Patients with a tracheostomy face complex changes in their self-management to adjust to their “daily life with the tracheostomy”. They are required to manage a multitude of changes in the context of “managing their therapeutic regimen”, “managing role and behavior changes”, and “managing their emotions”. The self-management model provided by Lorig and Holman [20] was adapted for patients with a tracheostomy; however, evidence on these three major dimensions differs widely. The majority of publications emphasized the dimension “management of the therapeutic regimen”, and “managing role and behavior changes”, however patient perceptions and skills required to care for the tracheostomy were reported to a lesser extent. In addition, the dimension “managing emotions” was investigated to a lesser extent in the articles studied. Aspects within the three dimensions overlap, e.g. considerations in the physical/ bodily component lead to role changes and/ or emotional implications.

Patients with a tracheostomy in the home setting have received considerable attention by the scientific community during the last decade. Most of the included articles studied patients with a permanent tracheostomy after laryngectomy, which leads to lifelong changes in self-management. These patients are facing a chronic oncological condition. Thus, the promotion of self-management is emphasized with a view to autonomy, adaptation and prevention of complications [72]. Jansen and colleagues [54] assume that self-management interventions for patients after laryngectomy can prevent the development of more serious complications, which may result in more intensive and costly interventions. Patients with head and neck cancer were the main population included in that study. They may suffer from various symptoms and side effects, which influence their physical and emotional wellbeing and quality of life [73, 74]. Symptom experiences and subsequent self-management may relate to the tracheostomy or to the underlying diagnoses, co-morbidities, or side effects of the treatment plan.

For the first self-management dimension, **managing the therapeutic regimen**, two sub-dimensions were identified: tracheostomy care and tracheostomy- related physical/ functional changes. Tracheostomy care was reported to a lesser extent (n = 6), although correct performance of that skill is essential for patient safety. Moreover, most of the included publications studied patients with respect to their self-management skills in the long-term and reported on participants who had the tracheostomy surgery on average more than six months prior to data collection (n = 24; 71%). Given the theoretical framework of self-management outlined by Lorig and Holman [20], patients’ self-management skills are time dependent. In the context of tracheostomy care, Wulff and associates [16] pointed out that how and when effects are measured is important. Previous studies have found that skills deteriorate right after laryngeal or hypopharyngeal cancer treatment and with subsequent tracheostomy surgery, and stabilize after approximately one year. In addition, the patient perspective with respect to prioritizing their self-management tasks and skills might shift over time [5, 75, 76] and requires further investigation. More precisely, Leemans and colleagues [77] concluded in a study with 1,705 laryngectomized patients that pulmonary changes in particular caused limitations in daily activities and social living.

The second self-management dimension, **managing role and behavior changes**, included the sub-dimensions of changed roles and changed everyday behavior. Overall, 47% of the studies (n = 17) reported on changed roles. Changes in self-identity, social and professional life were described; however, the implications of these changes on self-management in daily life were reported to a lesser extent. More studies (71%, n = 24) described changed everyday behavior. Dooks and colleagues [3] emphasized that day to day challenges were found by the patients to be more important when compared with the physical management after hospital discharge. Changes regarding social participation, sports activities / leisure activities, communication and eating indicated how profound these influences are on everyday behavior. Responses to these challenges may vary significantly by the individual. Considerable emphasis also has been given to changes in communication. In a study by Danker and associates [32], 54% of patients with tracheostomy talked less than before treatment, 57% stated that much was remaining unsaid, 51% exclusively talked about important things, 42% spoke as little as possible, and 40% refused to speak. Other important changes regarding eating and body care have been investigated to a lesser extent and need further exploration.

The third self-management dimension, **managing emotions**, can be characterized as adapting and coping with emotional changes having a tracheostomy. Overall, 47% of the included studies (n = 16) reported on a variety of emotions with an overwhelming proportion being negative emotions. Future research is needed with a focus on **managing emotions** throughout the healthcare experience following a tracheostomy. Continued psychosocial assessment, beginning in the pre-operative period and continuing to the long-term follow-up when patients have returned to the community setting [78] are needed in order to provide appropriate emotional and psychosocial support [8, 72, 76, 78, 79].

The majority of studies focused exclusively on patients after laryngectomy as a permanent condition, whereas a minority of four articles studied patients with a temporary tracheostomy. Although Everitt [78] argued in her expert statement that tracheostomy care does not differ for a temporary tracheostomy or a permanent tracheostomy, Querós and colleagues [31] suggested that the duration of the tracheostomy and the type of surgery are relevant influencing factors on self-management. Future research of self-management of patients with tracheostomy must take this aspect into account.

### Strengths and limitations

This study carries several strengths and limitations. First and foremost, a strength of this study is that it was based on the theoretical framework, the definition and the operationalization of the concept of self-management [41]. Because self-management is often used in the context of chronic conditions [17], the applicability of generally accepted self-management definitions and concepts was critically examined in advance. Such definitions must be refined to situation- and/ or disease specific settings [41], and then put into the context of the patient population under investigation. In addition, the data analysis presented provided further description and specification of the dimensions and sub-dimensions of the concept of self-management of patients with a tracheostomy.

This review included studies with adults (> 18 years of age) with either a temporary or permanent tracheostomy, who were fully or partially self-sufficient in tracheostomy care and lived at home. Studies, which did not clearly describe these characteristics were excluded from this analysis, which may have resulted in important data being overlooked. For the literature search, four major databases were used. However, potentially relevant sources might have been missed in databases not considered for this study. Also, articles in languages/ idioms not available to the reviewer might limit our findings. Furthermore, the process of study selection, abstraction and analysis was performed by one person, critically appraised, and consented by the work group and might have led to a potential risk of bias. In addition, it can be assumed that internal and external factors at the individual or micro level may influence self-management processes [80], which also was not the focus of this review. Finally, the aim of this study was an overview of existing literature on self-management of patients with tracheostomy. Since a scoping review does not require a quality assessment [36], included studies had their own strengths and limitations.

## Conclusions

This scoping review represents the first comprehensive overview of patient-centered outcomes with respect to the complex self-management tasks and skills required of patients with a tracheostomy living at home. Three self-management dimensions - “managing the therapeutic regimen”, “managing roles and behavior change”, and “managing emotions” - were identified and systematically integrated into a theoretical model. This model can serve as a cornerstone for empirical intervention studies to better support this patient population in the future. A multi-professional approach is recommended to best meet the needs of these patients in their home setting.

### Electronic supplementary material

Below is the link to the electronic supplementary material.

Supplementary Material 1

Supplementary Material 2

## Data Availability

All data analysed and synthesized during this study are included in this published article.
