# Supplementary material for: Self-management of patients with tracheostomy in the home setting: a scoping review
Source: J Patient Rep Outcomes. 2023 Oct 12;7:101. doi: 10.1186/s41687-023-00643-2 (PMC10570259; doi:10.1186/s41687-023-00643-2)
Supplement: Supplementary file 2 — Supplementary Material 2 [file 41687_2023_643_MOESM2_ESM.docx]

Journal of Patient-Reported Outcomes

Self-management of patients with tracheostomy in the home setting: A scoping review

**File 1** Search terms (Medline)

| Participants (P) | | |
| --- | --- | --- |
| Tracheostomized patients | #1 | “tracheostom*” OR “tracheotom*” OR “laryngectomy*” |
|  | #2 | (“care” OR “caring”) AND “tube” |
|  | #3^a^ | Tracheostomy/  Tracheotomy/  Laryngectomy/ |
| Concept (C) | | |
| Self-management (C1) | #4 | “self-mange*” OR “self manage*” OR “self care” OR “self efficacy” OR “self regulat*” OR “self mainte*” OR “self monitor*” OR “self observ*” OR “self concept” OR “self care abilit*” OR “self care agenc*” OR “self care manage*” OR “self care mainte*” |
|  | #5^a^ | Self-Management/  Self Care/  Self Efficacy/  Self Concept |
|  | #6 | “patient education” |
|  | #7^a^ | Patient education as Topic/ |
| Effects of tracheostomy (C2) | #8 | “activities of daily living” OR “quality of live” OR “well being” OR “body image” OR “stigma” OR “adaptation” OR “adjustment” OR “coping” |
|  | #9^a^ | Activities of Daily Living/  “Quality of life”/  Body Image/  Social Stigma/  Adaptation, Psychological/  Social Adjustment/ |
| Combination | | |
| Combine 1 | P | #1 OR #2 OR #3 |
| Combine 2 | C1 OR C2 | #4 OR #5 OR #6 OR #7 OR #8 OR #9 |
| Combine 3 | P AND C | Combine 1 AND Combine 2 |
| ^a^MESH terms | | |
